# On the Preparation of Pyrophosphate of Iron

**Published:** 1869-07

**Authors:** Samuel P. Duffield


### ON THE PREPARATION OF PYROPHOSPHATE OF IRON.

By SAMUEL P. DUFFIELD, PH. D.

QUERY No. 26. It is found that the process of the Pharmacopœia for pyrophosphate of iron yields a preparation which it is sometimes impossible to scale. Can a better process be devised?

Pyrophosphate of iron rendered soluble by means of citrate of ammonia was first proposed by M. E. Robiquet, to the Academy of Medicine at Paris. As prepared by him, the gelatinous precipitate was dissolved in citrate of ammonia solution, and a syrup was made from this solution. This process was improved by Prof. Proctor; the result was also a syrup. The formula, as it occurs at present in the United States Pharmacopœia, is that of Dr. E. R. Squibb, and exhibits the finished product in scales.

The pharmacopœia process is to ignite the $2NaO, HO, PO_5 + 24HO$, thereby converting it into the $2NaO, PO_5$. This is dissolved in water and mixed with solution of tersulphate of iron, at a temperature not exceeding 50° Fahr.; the resulting pulpy precipitate washed thoroughly and dissolved in solution of citrate of ammonia.

This process I have been disappointed in; it scales but imperfectly, and sometimes not at all.

I am not alone in my complaint; several have tried the same process and failed to get satisfactory results. After having tried faithfully for four times this process, I devised a slight modification, which gave me the most satisfactory results, never failing once to scale handsomely.

The process:—Take of the magma obtained from 8½ oz. of pyrophosphate of soda (which is about 6½ times as much by weight)—

| | | |
|---|---|---|
| Aqua ammoniæ. | - - - - | 1 pint. |
| Citric acid, | - - - - - - | 6 oz. |

Mix the pyrophosphate of iron (the magma) with the ammonia, and digest at a gentle water-bath heat, for 6 to 8 hours; then *gradually* add, constantly stirring, the 6 oz. of citric acid, dissolved in two pints of distilled water, *until* the ammonia is

*neutralized*, and the precipitate dissolved. Filter or decant, if necessary; evaporate to the consistency of a thick syrup; paint on glasses and scale as usual. The change which takes place on the addition of the ammonia is different from the appearance produced by the addition of citrate of ammonia solution.

In the case of the addition of aqua ammoniæ, the magma turns, in a few hours, of brick-red color; and in the case of the addition of citrate of ammonia solution, it dissolves to a gum color, similar to the iodide of iron liquor, before adding the syrup.

If, now, you add the citric acid to the brick-red colored magma, it gradually dissolves and becomes a greenish color by transmitted light, and dark-brown, almost black, when concentrated by reflected light.

That there is a different apportioning of elements produced by the modification, no one can deny. In the first you have pyrophosphate of iron in solution, by means of citrate of ammonia, a mixture of neutral salts. In the second you have, on the addition of ammonia, pyrophosphate of ammonia and sesquioxide of iron, which latter exists in a free state. On the addition of the citric acid it unites with the sesquioxide of iron, forming the citrate of the sesquioxide, mixed with the phosphate of ammonia. I think from this it will be seen that the composition is changed, the iron in the pharmacopœia formula (Squibb's) existing as a pyrophosphate; the iron in my modification existing as a citrate of the sesquioxide.

As the Pharmacopœia Committee consider their preparation an intimate admixtrue of ferruginous and ammoniacal salts, I do not consider I have *violated* the U. S. P.

DETROIT, *Aug. 5, 1868.—Proceedings of American Pharmaceutical Association, at 16th Annual Meeting, held Sept., 1868.*

